# The Use of Antidepressants, Anxiolytics, Sedatives and Hypnotics in Europe: Focusing on Mental Health Care in Portugal and Prescribing in Older Patients

**DOI:** 10.3390/ijerph17228612

**Published:** 2020-11-19

**Authors:** Marta Estrela, Maria Teresa Herdeiro, Pedro Lopes Ferreira, Fátima Roque

**Affiliations:** 1 iBiMED—Institute of Biomedicine, Department of Medical Sciences, University of Aveiro, 3800 Aveiro, Portugal; teresaherdeiro@ua.pt; 2 Centre for Health Studies and Research (CEISUC), 3000 Coimbra, Portugal; pedrof@fe.uc.pt; 3 Faculty of Economics, University of Coimbra, 3000 Coimbra, Portugal; 4 Research Unit for Inland Development, Polytechnic of Guarda (UDI-IPG), 6300 Guarda, Portugal; froque@ipg.pt; 5 Health Sciences Research Centre, University of Beira Interior (CICS-UBI), 6200 Covilhã, Portugal

**Keywords:** elderly, health policy, mental health, older patients, antidepressants, anxiolytics, sedatives and hypnotics, Portugal, Europe

## Abstract

(1) Background: Mental disorders are a growing concern in the 21st century. The most prevalent common mental disorders include depression and anxiety. It is predicted that half of the population will at some point in their lives experience one or more mental disorders. Although common mental disorders are highly prevalent, some of the most significant related problems are the wide treatment gap and the excessive use of antidepressants, anxiolytics and sedatives/hypnotics, especially among older patients. (2) Methods: This study aimed to analyze mental health care in Portugal, with a focus on the consumption of antidepressants, anxiolytics, sedatives and hypnotics among older patients. (3) Results: The use of antidepressants, anxiolytics, sedatives and hypnotics has increased overall across Europe. In Portugal, a downward trend of sedatives and hypnotics consumption can be observed. Anxiolytics and antidepressants, on the other hand, have been increasing. Patients aged ≥60 years old consume more than half of the aforementioned drugs. (4) Conclusions: Mental health policies should be designed to improve the conscientious use of antidepressants, anxiolytics, sedatives and hypnotics, particularly among older adults.

## 1. Introduction

Mental health has been a major concern in the 21st century. In fact, mental health-related disorders are the non-fatal disorders with the greatest impact globally, accounting for more than 7% of the global burden of disease, and one-third of the burden of disease in Europe [1]. Despite the increasing attention paid to global mental health in recent years, along with the publication of guidelines [2,3] for the prevention and treatment of mental disorders, the transposition into real-world benefits for those with mental health problems has been slow. The impact of mental disorders worldwide is highly prominent: about one billion people are affected by mental disorders or substance abuse worldwide [4]. The economic impact is also severe: it is estimated that a loss of 16 trillion US$ caused by mental disorders is faced by the global economy when considering the productivity losses throughout the course of lives and the early onset of mental health disorders [5].

The gap between the need for treatment for mental disorders and its provision is visible around the globe. In low and middle-income countries, between 76% and 85% of people with severe mental disorders do not receive treatment for their mental health problems. The interval corresponding to high-income countries is also high, at between 35% and 50% [6]. 

Another problem is related to the considerable access gap to timely care, both in terms of diagnosis and follow-up. Although the prevalence of patients with mental disorders is more than 20%, less than 10% of Portuguese patients registered in primary health care are diagnosed with depressive disorders or anxiety. Only 35% of patients with any type of mood disorder had an appointment in the same year as the onset of the disorder, corresponding to a median delay of five years, and only 37.8% of those with major depression received any kind of treatment in the year of the onset of the disorder, which corresponds to a median delay of four years [7]. 

In Europe, mental disorders are by far the main contributors to chronic conditions affecting the population. According to the latest available data, neuropsychiatric disorders are the leading cause of years lived with disability (YLD) in Europe, corresponding to 36.1% of those attributable to all causes. Unipolar depressive disorder accounts for 11% of all YLD, making it the leading chronic condition in Europe. Anxiety disorders are in sixth place, accounting for 4% of all YLD [8].

It is estimated that by 2030, unipolar depressive disorders will be the leading cause of burden of disease globally. Consequently, mental disorders are a major challenge for health systems, as they are very prevalent and represent more than €460 billion in direct costs in Europe [1]. The prevalence of diagnosed mental disorders in Portugal is the highest in Europe, and mood and anxiety disorders are the most common of these. Mental disorder in Portugal also implies societal costs associated with days “out-of-work” that are far above those of other high-income countries, with 20.2% of unproductive days in a month being due to mental disorders [1,9].

Portuguese data from 2015 show that less than 0.8% of the total expenditure for health is allocated to mental health care, making Portugal one of the countries with the lowest funding for this type of care in the EU-27 [10]. Considering that less than 10% of the total Portuguese gross domestic product (GDP) is allocated to health—with this percentage also being lower than the Organization for Economic Cooperation and Development (OECD) average—the chronic underfinancing of mental health care is evident [11]. 

The current Mental Health Law (no. 36/98) was approved in 1998—more than 20 years ago—establishing the general principles of mental health policy and regulating the compulsory internment of people with mental health disorders [12]. It was only then that the need to create a diverse and articulated network with inter-ministerial collaboration and community social organizations was officialized, which has fostered a discussion of the system’s contradictions and weaknesses [12]. Alongside the growing concern regarding mental health care, in 2006, a National Commission for the Restructuring of Mental Health Care was created. Its purpose was not only to ascertain the situation of the provision of mental health care but also to create a plan to restructure and develop mental health services. In 2007, the National Mental Health Plan 2007–2016 (NMHP)—now extended until 2020—emerged. This is aligned with the guidelines of the World Health Organization (WHO) and other international organizations with action on mental health issues. The values of this plan consist mainly of assuring that mental health remains inseparable from general health. It also defends the preference for mental health care in the community setting, the protection of the human rights of those who suffer from mental disorders and the coordination and integration of care [13]. 

Despite the existence of the NMHP and other European and international strategies, 2018 reports show that, in Portugal, the 12-month prevalence of mental disorders is 21.0%, constituting a particularly high prevalence when compared to other Southern European countries such as Italy and Spain, with 12-month prevalence rates of 9.7% and 8.8%, respectively [1,14]. Evaluation reports of NMHP identify the constraints that explain, at least to some extent, the obstacles associated with the improvement of mental health care and the aforementioned numbers [13]. 

Although NMHP has been implemented for over 10 years, chronic problems are still being highlighted, namely the low autonomy and decision-making capacity of those responsible for implementing it and local decision-making centers. This compromises the improvement of care in the community as a result of a lack of coordination, the great concentration of human resources only in the central hospitals and inadequate models of financing and management [13]. Nevertheless, Portugal is considered a reference country in terms of mental health policies by three institutions: the EU Joint Action on Mental Health and Wellbeing (JA MH-WB), the UN Commission on Human Rights and the Global Platform Gulbenkian Mental Health [15]. 

Besides the difficulties associated with mental disorders, poor access and the underfinancing of the mental health care system, one of the biggest problems is the excessive consumption of antidepressants, anxiolytics, sedatives and hypnotics [16]. OECD statistics from 2015 show that the volume of sales of anxiolytics in outpatient services accounted for 2.2% of all drugs sold in Portugal, which ranks first in comparison to all other 25 OECD countries. In terms of the consumption of hypnotics and sedatives, Portugal was in seventh place of the OECD-26 countries, with a sales volume of 0.8%. Regarding antidepressants, Portugal was third, with a sales volume standing at 3.7%, which was only surpassed by Canada and Spain [17]. 

The prescription of these drugs for older patients raises important concerns, as ageing and multimorbidity are usually associated with polypharmacy, where older patients take multiple medicines for long periods of their lives [18,19,20]. The use of multiple medications contributes to an inappropriate use of medicines, which can interfere with the effectiveness of treatments [18]. According to the American Geriatrics Society’s Beers Criteria for Potentially Inappropriate Medications in the Elderly, the prescription of these drugs for older patients should be avoided, as they are associated to several adverse reactions and potential dangers [21]. Antidepressants have high anticholinergic properties, causing sedation and orthostatic hypotension. On the other hand, sedatives and hypnotics present high rates of dependence and a great risk of overdose when low dosages are prescribed. Anxiolytics, especially benzodiazepines, are associated with cognitive impairment, fractures, falls and delirium. Furthermore, older adults have higher sensitivity to benzodiazepines and a decreased metabolism of long-acting benzodiazepines [21].

The rates of prescription, polypharmacy and recommendation of potentially inappropriate medication are very high in Portugal. As polypharmacy is one of the most important factors associated with potentially inappropriate medication, physicians should account for the risks of prescribing anxiolytics and antidepressants, which are highly consumed by this age group [20,22,23]. 

In Portugal, 65.5/1000 adults aged ≥65 years consume benzodiazepines chronically, representing the third-highest consumption rate among the OECD-17 countries and corresponding to almost double the OECD-17 average. The consumption of long-acting benzodiazepines by the elderly in Portugal ranks fifth among the OECD-18 countries, where more than 80/1000 adults aged ≥65 years consume these drugs despite the fact that they are not recommended for older people, as they have a decreased metabolism for this type of benzodiazepines, and, consequently, take longer to eliminate them from the body [20]. 

Considering that anxiolytics and antidepressants are highlighted as some of the most frequent prescribed drugs for older patients and that there is currently no evidence that shows a reduction of this consumption patterns [16,18], the current study aims to critically evaluate the mental health care framework, providing an analysis confronting both mental health policies and drug consumption in Portugal. By analyzing the consumption of antidepressants, anxiolytics, sedatives and hypnotics across Europe and Portugal, with a special focus on the older population, this study expects to (1) provide a critical perspective on the mental health framework in Portugal and (2) shed light on the main risks associated with the use of anxiolytics, antidepressants, sedatives and hypnotics by older people, while critically analyzing the policies designed and implemented for the improvement of prescription practices. 

## 2. Methods 

The scope of this study encompasses the drug classes corresponding to the ATC (Anatomical Therapeutic Chemical) codes N05B (anxiolytics), N05C (sedatives and hypnotics) and N06A (antidepressants). The drugs for each ATC Code are presented in Table S1. European data were retrieved directly from the OECD statistics database and represent the drugs dispensed from 2000 to 2018. The data in the OECD database were officially provided by each country [24]. The database consists of 26 countries. European countries listed in this database were selected by three criteria: (i) the use of the same or a similar data collection methodology, (ii) a small number of missing values in databases and (iii) the heterogeneity of the locations. Two countries were chosen from each of the four regions defined by EuroVoc: Western Europe (Germany and Luxembourg), Eastern Europe (the Czech Republic and Slovakia), Southern Europe (Portugal and Spain) and Northern Europe (Iceland and Sweden) [25]. 

Data regarding the Portuguese consumption were anonymously provided by the National Authority for Medicines and Health Products (INFARMED), representing the drugs dispensed on the National Health Service (NHS)-reimbursed market from 2000 to 2018. European and national data depicting the consumption of drugs corresponding to the ATC codes N05B, N05C and N06A were presented as the number of defined daily doses (DDDs) dispensed per 1000 inhabitants per day. Both databases were analyzed through graphical presentations and processed using the IBM SPSS v25 statistical software. Initially, exploratory and descriptive statistical analyses of the variables were applied to identify any missing values. In a subsequent phase, inferential statistical analysis techniques were applied. Descriptive and linear regression analyses were conducted for the Portuguese data. To verify the consistency of the regression analysis, the assumptions of linearity, normality, autocorrelation and homoscedasticity were verified. The current study is a cross-sectional and retrospective observational study.

Data concerning consumption by gender and age were retrieved from an INFARMED [26] study and represent the anxiolytics, sedatives, hypnotics and antidepressants that were dispensed in community pharmacies in mainland Portugal. These drugs were classified according to a systematization according to their identity and the therapeutic indications for which they have been approved and authorized [27]. As the national drug classification merges N05B and N05C into one category, the data obtained are thus presented as two main groups: anxiolytics, sedatives and hypnotics and antidepressants. 

## 3. Results

### 3.1. Use of Antidepressants, Anxiolytics, Sedatives and Hypnotics in Europe

**Figure 1:**
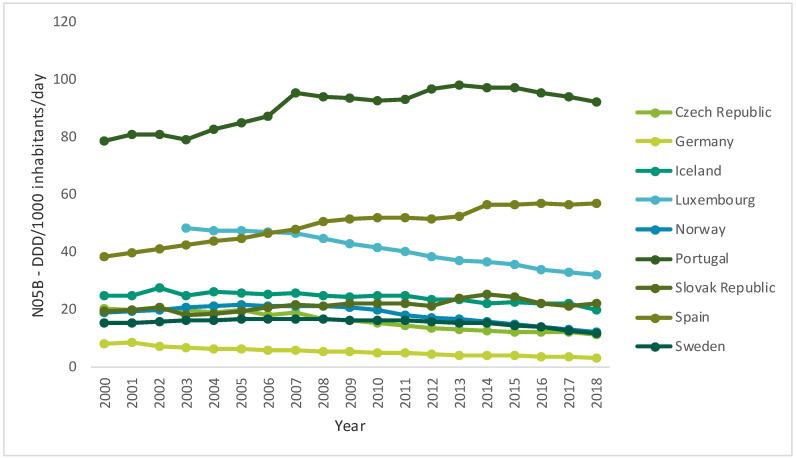
Use of anxiolytics (N05B) in some Organization for Economic Cooperation and Development (OECD) countries from 2000 to 2017 (DDD/1000 inhabitants/day) (data from [24]).

**Figure 2:**
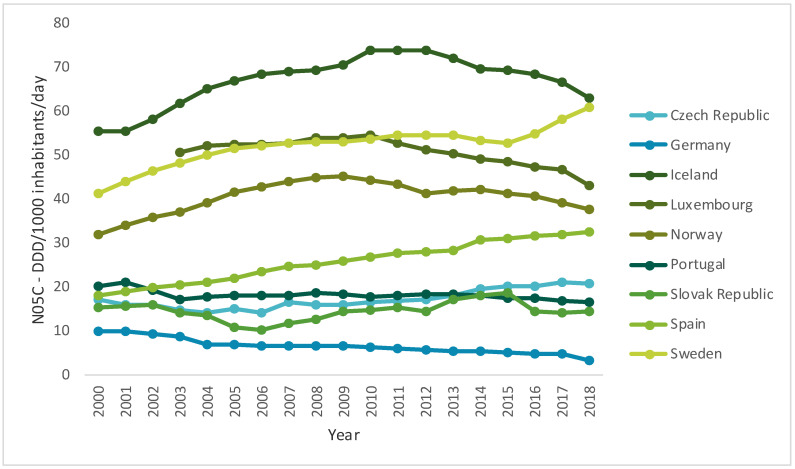
Use of sedatives and hypnotics (N05C) in some OECD countries from 2000 to 2017 (DDD/1000 inhabitants/day) (data from [24]).

**Figure 3:**
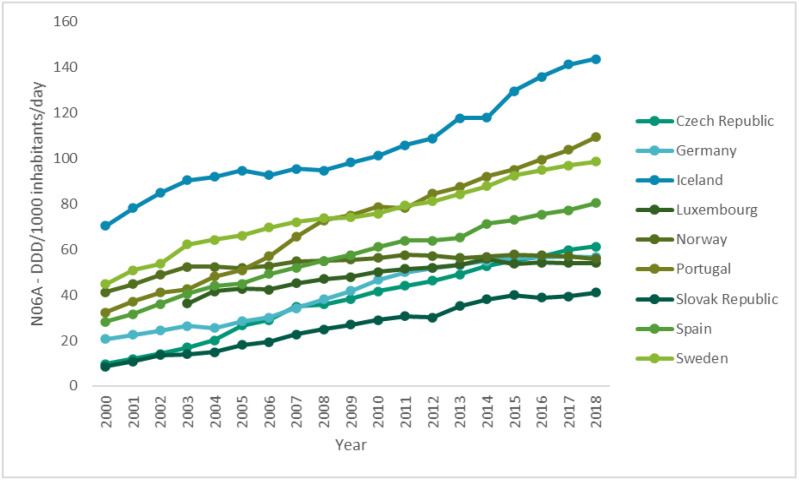
Use of antidepressants (N06A) in some OECD countries from 2000 to 2017 (DDD/1000 inhabitants/day) (data from [24]).

Over time, the use of antidepressants, anxiolytics, sedatives and hypnotics has increased across Europe. However, a slight decrease in the consumption of anxiolytics in almost all countries in the past few years has been observed (Figure 1) [24]. However, despite the recommendations, the consumption of benzodiazepines remains excessive, and the prescriptions of this kind of drug are maintained for periods greater than recommended—six months—in different parts of the world, especially in developed Western countries [16]. On the other hand, while the consumption of sedatives and hypnotics tends to be more heterogeneous (Figure 2), all countries have witnessed a very pronounced increase in the consumption of antidepressants (Figure 3) [24]. 

### 3.2. Consumption of Antidepressants, Anxiolytics, Sedatives and Hypnotics in Portugal

Regarding the descriptions of anxiolytics, antidepressants, sedatives and hypnotics in terms of DDDs consumed from 2000 to 2018, it is observed that anxiolytics (N05B) (μ = 60.15; σ = 6.35) and antidepressants (N06A) (μ = 58.45; σ = 27.93) present quite similar mean values, although the standard deviation of the latter is much higher than the other two classes. The use of hypnotics and sedatives (N05C) over time has an average consumption rate that is much lower than those corresponding to anxiolytics and antidepressants (μ = 10.36; σ = 1.01).

**Figure 4:**
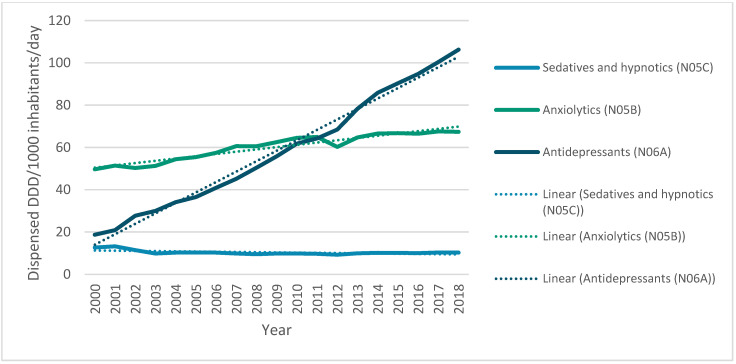
Use of antidepressants, anxiolytics, sedatives, and hypnotics in Portugal from 2000 to 2018 (DDD/1000 inhabitants/day).

Figure 4 depicts the use of antidepressants, anxiolytics, sedatives and hypnotic drugs in Portugal from 2000 to 2018. A very pronounced increase in the consumption of antidepressants has been observed. In 2000, it did not exceed 20 DDD/1000 inhabitants/day, while in 2018 it reached almost 110 DDD/1000 inhabitants/day. The line defined by the consumption of anxiolytics also shows a growing trend, although it is possible to observe a slight decrease in early 2011 and a subsequent increase by 2012. The use of sedatives or hypnotics, in contrast, shows a slight decrease at the beginning of the millennium and a trend towards stagnation from that point on. 

After the descriptive analysis of the data, the linear regression analysis was completed. It was found that the three variables follow the autocorrelation assumptions of linearity, homoscedasticity and normality, except for the hypnotics and sedatives (N05C) variable, which did not obey the linearity assumption (see Supplementary Material). The results obtained by the linear regression analysis show different slopes regarding the tendencies of consumption: both anxiolytics (N05B) and antidepressants (N06A) present a very pronounced, significant (*p*-value < 0.001) and positive slope (B = 0.954 and B = 0.994, respectively), while for hypnotics and sedatives (N05C), the slope obtained was negative and not as pronounced as the former drug classes (B = −0.566), although it was also statistically significant (*p*-value = 0.005). It is possible to infer that the consumption of anxiolytics and antidepressants has increased and to assume a decreasing trend of the consumption of sedatives and hypnotics in Portugal.

### 3.3. Consumption of Antidepressants, Anxiolytics, Sedatives and Hypnotics in Portugal among Older Patients

The table below (Table 1) depicts the number of patients that have consumed antidepressants, anxiolytics, sedatives and hypnotics by age and sex in mainland Portugal. 

It is observed that, in all cases, the proportion of people that consume these drugs aged ≥60 years corresponds to more than 50% of the total. The highest consumption corresponds to patients with ages between 60 and 79 years old. It is also observed that, overall, women consume more than twice as much of both drug classes than men.

## 4. Discussion

### 4.1. Consumption of Antidepressants, Anxiolytics, Sedatives and Hypnotics around Europe

According to OECD.stats, it appears that the use of psychotropic drugs has increased over time. Nevertheless, the consumption of anxiolytics has decreased in almost all countries, except for Slovakia, Spain and Portugal [24]. This phenomenon may result from anxiety drugs being sold at a lower price, either due to the widespread availability of more affordable generic drugs or because of the low investment in therapeutic innovation [28,29]. This also agrees with the data reported on the European Social Survey, which show a high risk of serious depressive symptoms in Southern and Central and Eastern Europe, which may also be related to the lower life conditions in these countries and lack of social support [30].

Regarding the consumption of sedatives and hypnotics, overall consumption has increased in Europe, which can be explained by the increasing prevalence of sleep disorders or by the increase of the prescriptions of these drugs [31,32].

Regarding the use of antidepressants, there is a very pronounced increase in the consumption of this pharmacotherapeutic group in all countries, which is associated with a decrease in the proportion of the budget resources available for these drugs to the total budget. This phenomenon could be explained by several factors: the increased prevalence of common mental disorders, antidepressant prescription over non-pharmacological therapies, growing access to antidepressants or low investment in therapeutic innovation [33].

The consumption of antidepressants has increased noticeably in the last decade, especially in higher-income countries. Part of this growth results from the increasing intensity and duration of treatment and from the higher number of therapeutic indications, which has raised concerns about the appropriateness of prescriptions [30]. One of the reasons for this recent increase is the emergence of the 2008 economic crisis, which is reflected in an increase of the insecurity and instability of the individuals. However, the consumption of antidepressants has increased to an even greater extent in countries such as Germany, which were less affected by the economic crisis, with a growth of 46% between 2007 and 2011 [34].

### 4.2. Consumption of Antidepressants, Anxiolytics, Sedatives and Hypnotics in Portugal

The increased consumption of anxiolytics and antidepressants may be due to the prolonged use of these drugs, to the existence of more new users because of a growth in the diagnostic rate, to greater access to these drugs or to a rise in therapeutic indications [35]. Since taking anxiolytics for prolonged periods can cause addiction, it is possible that part of the increase in DDD prescribed over the years results from a cumulative effect, revealing the existence of users who take anxiolytics for longer periods than was prescribed. It should also be noted that the Portuguese General Directorate of Health 2017 report shows that, although there was a decrease of 46.2% in NHS charges for the packaging of these drugs, there was an increase in the number of boxes sold on the order of 97.3% and thus in the total charge to the NHS [36].

A major problem in Portugal is the high dispensing rate of benzodiazepines despite the risks of serious adverse effects. The prescription of these anxiolytics is essentially carried out in primary health care, especially in the northern and central regions of the country [37]. However, observing the trend in the consumption of anxiolytics since 2013, there has almost been a stagnation. Since benzodiazepines are the most widely used anxiolytics in this therapeutic class, this stagnation might be explained by some measures that have been implemented to halt the consumption of benzodiazepines, especially the development of a withdrawal protocol for chronic benzodiazepine use and the publication of guidelines for the treatment of anxiety disorders [38,39].

However, it was still observed that, since 2002, there has been a sharp increase in the consumption of antidepressants, which may have been caused by the enlargement of the special reimbursement scheme for antidepressants prescribed by physicians other than psychiatrists. The co-payment rate of these drugs by the Portuguese NHS, which had been 40%, increased to 70%. Antidepressants thus became accessible to a wider range of the population [35]. However, although the co-payment rate of antidepressants changed to 37% since 2010, their consumption continues to register a significant growth trend [35]. Only surpassed by viral and bacterial vaccines and anti-inflammatory drugs, antidepressants constitute the fourth largest pharmacological class associated with adverse drug reaction reports in Portugal, with approximately 5% of all reports being in the central region of the country according to a 2015 study reporting the cases from 2000 to 2013. Furthermore, although anxiolytics, sedatives and hypnotics represent less than 1.5% of all adverse drug events combined, it is important to note the high prevalence of side effects for these drug classes. [40] More recently, INFARMED’s authorities report that psycholeptics, which include anxiolytics, sedatives and hypnotics, account for 4% of all reported adverse drug reactions in Portugal [41,42].

### 4.3. Mental Healthcare Policies in Portugal Associated with the Consumption of Antidepressants, Anxiolytics, Sedatives and Hypnotics: Focusing on Older Patients

Regarding policies related to the use of antidepressants, anxiolytics, sedatives and hypnotics in Portugal, public health bodies have paid a great deal of attention to the consumption of benzodiazepines. The protocol for withdrawal from the chronic use of benzodiazepines, developed by the Institute of Preventive Medicine and Public Health of the Lisbon Medical School with the aim of evaluating interventions to discontinue the chronic use of benzodiazepines in the context of primary health care, was a considerable step towards the reduction of the use of this type of drug [38].

In 2017, the symposium “Sleep and relax without being dependent on benzodiazepines” took place, and it was promoted by the Coordination of the National Strategy of Drug and Health Products. This campaign sought to raise awareness in health professionals and citizens about the excessive use of benzodiazepines [43]. It was observed that, over time, awareness campaigns have been implemented to tackle the excessive use of benzodiazepines [43]. Due to the risk of dependence and other important side effects, these campaigns may have been reflected in benzodiazepine consumption, which has tended to stagnate for nearly 10 years. The decrease in the use of anxiolytics in several European countries may also be reflected in the increase in mental health care investment, enhanced literacy and the effect of some preventive actions associated with non-pharmacological models to support those suffering from anxiety disorders [44]. Regarding antidepressants, however, the very pronounced increase in consumption has only very recently been followed by any political action to halt this trend [45].

It is important to note that the consumption of antidepressants, anxiolytics, sedatives and hypnotics is very pronounced among older patients, especially in women. Polypharmacy is very prominent among the older Portuguese population, as is the risk of potentially inappropriate medication [23]. Despite the risks associated with polypharmacy and benzodiazepine use, benzodiazepines are often prescribed for older adults that suffer from anxiety and sleep disorders. Moreover, the fact that Portugal is one of the countries with the highest level of chronic benzodiazepine use should raise serious concerns, as these drugs create dependency when used for periods greater than 2 to 4 weeks and are associated with significantly higher risks of fractures, falls and cognitive deficits [20,43]. Furthermore, although the literature suggests that older adults do not exhibit a response or remission of depressive symptoms, antidepressants are widely used among frail older patients [46].

High levels of consumption of antidepressants, anxiolytics, sedatives and hypnotics can be explained by poor access to non-pharmacological treatments in the NHS. This might be caused by the chronic underfunding of the health sector, especially in mental health. This underfunding has a direct impact on the number of mental health professionals, which is particularly low in Portugal. In fact, the ratio of mental health providers is 25/100,000 inhabitants, which is at the lower end of the range according to the European standards, requiring careful consideration of how to achieve an optimal balance between community service teams and hospitals [47].

### 4.4. Conclusions

The aforementioned factors influence the quality of mental health services, hindering access to care and the proper treatment of individuals suffering from mental disorders. However, the problems associated with the mental health paradigm in Portugal are not restricted to funding, structural and organizational issues. It is also important to invest in mental health literacy, particularly in schools and at the workplace. This investment will contribute to the reduction of stigma and promote the demand for mental health care by those who suffer from mental disorders, which would result in a decrease in the treatment gap, medication rates and possibly drug use [13].

Considering the high prevalence of mental disorders and their associated problems, it is evident that there is a need to make political decisions to improve mental health care. In this respect, the measures adopted by various organizations, especially the WHO worldwide, the JA MH-WB at the European level and the Directorate-General of Health at the national level, have a major impact not only on improving the mental health paradigm but also on stimulating discussion with various agencies and society regarding this topic [48,49,50].

This study presented an analysis of the consumption of antidepressants, anxiolytics, sedatives and hypnotics in Portugal and related them to mental healthcare policies. However, it is crucial to emphasize that the data obtained are exclusively related to the outpatient reimbursed market. As such, all drugs dispensed in hospitals are not considered, nor are those that are fully paid by the consumer. However, we believe that the inclusion of these data would not alter the interpretation of results. Additionally, it is important to note that these figures are an estimate of actual consumption; i.e., the volume of drugs dispensed to consumers is quantified, which does not necessarily mean that consumers actually take these drugs.

Mental health is an area that should be addressed in depth in an integrated manner with other specialties and health sectors. The improvement of prescribing practices—one of the easiest issues to monitor and control—could be a starting point for improving treatments of mental disorder, particularly concerning common mental disorders such as depression and anxiety. Additionally, policies regarding mental health should focus on decreasing the treatment gap and addressing the mental health of the population, both in society in general and in institutions such as schools and workplaces. Further research is suggested regarding the use of antidepressants, anxiolytics, sedatives and hypnotics, especially among older adults, considering a multiplicity of approaches, namely consumption patterns, the associated risks and policy issues.

## Figures and Tables

**Table 1:** Anxiolytics, sedatives, hypnotics and antidepressants dispensed by age and sex in Portugal.

	Anxiolytics (N05B) and Sedatives and Hypnotics (N05C)				Antidepressants (N06A)			
	Men		Women		Men		Women	
Age	*n*	% of Total	*n*	% of Total	*n*	% of Total	*n*	% of Total
0–19	10,059	1.65%	15,329	1.17%	8047	1.85%	13,995	1.17%
20–39	71,706	11.77%	129,889	9.88%	58,132	13.36%	128,400	10.70%
40–59	195,976	32.17%	416,738	31.69%	137,667	31.64%	411,375	34.29%
60–79	249,012	40.87%	531,409	40.41%	167,332	38.45%	464,181	38.70%
≥80	82,520	13.54%	221,574	16.85%	63,990	14.70%	181,618	15.14%
Total	609,273		1,314,939		435,168		1,199,569	

## Supplementary Materials

The following are available online at https://www.mdpi.com/1660-4601/17/22/8612/s1, Table S1: Drugs corresponding to each Anatomical Therapeutic Chemical (ATC) code, Table S2: Autocorrelation test output (N05B, N05C, and N06A), Figure S1: Linearity test output (zresid*zpred)—N05B, Figure S2: Linearity test output (zpred*dependnt)—N05B, Figure S3: Linearity test output (zresid*zpred)—N05C, Figure S4: Linearity test output (zpred*dependnt)—N05C, Figure S5: Linearity test output (zresid*zpred)—N06A, Figure S6: Linearity test output (zpred*dependnt)—N06A, Figure S7: Homocedasticity test output (sresid*zpred)—N05B, Figure S8: Homocedasticity test output (sresid*zpred)—N05C, Figure S9: Homocedasticity test output (sresid*zpred)—N06A, Table S2: Normality test output (N05B, N05C, and N06A).
