# Supplementary material for: The Use of Antidepressants, Anxiolytics, Sedatives and Hypnotics in Europe: Focusing on Mental Health Care in Portugal and Prescribing in Older Patients
Source: Int J Environ Res Public Health. 2020 Nov 19;17(22):8612. doi: 10.3390/ijerph17228612 (PMC7699589; doi:10.3390/ijerph17228612)
Supplement: Supplementary file 1 [file ijerph-17-08612-s001.pdf]

Supplementary material

Table S1. Drugs corresponding to each ATC code.

ATC Code	ATC code/Drug name
N05B	N05BA01 diazepam
	N05BA02 chlordiazepoxide
	N05BA03 medazepam
	N05BA04 oxazepam
	N05BA05 potassium clorazepate
	N05BA06 lorazepam
	N05BA07 adinazolam
	N05BA08 bromazepam
	N05BA09 clobazam
	N05BA10 ketazolam
	N05BA11 prazepam
	N05BA12 alprazolam
	N05BA13 halazepam
	N05BA14 pinazepam
	N05BA15 camazepam
	N05BA16 nordazepam
	N05BA17 fludiazepam
	N05BA18 ethyl loflazepate
	N05BA19 etizolam
	N05BA21 clotiazepam
	N05BA22 cloxazolam
	N05BA23 tofisopam
	N05BA24 bentazepam
	N05BA56 lorazepam, combinations
	N05BB01 hydroxyzine
	N05BB02 captodiame
	N05BB51 hydroxyzine, combinations
	N05BC01 meprobamate
	N05BC03 emylcamate
	N05BC04 mebutamate
	N05BC51 meprobamate, combinations
	N05BD01 benzoctamine
	N05BE01 buspirone
	N05BX01 mephenoxalone
	N05BX02 gedocarnil
	N05BX03 etifoxine
	N05BX04 fabomotizole
	N05BX05 Lavandulae aetheroleum
N05C	N05CD08 midazolam
	N05CF03 zaleplon
	N05CF04 eszopiclone
	N05CH01 melatonin
	N05CH03 tasimelteon
	N05CM18 dexmedetomidine
N06A	N06AB03 fluoxetine
	N06AB04 citalopram
	N06AB05 paroxetine
	N06AB06 sertraline
	N06AB08 fluvoxamine
	N06AB10 escitalopram
	N06AX11 mirtazapine
	N06AX12 bupropion
	N06AX16 venlafaxine
	N06AX17 milnacipran
	N06AX18 reboxetine
	N06AX21 duloxetine
	N06AX22 agomelatine
	N06AX26 vortioxetine
	N06AX27 esketamine

Table S2. Autocorrelation tests output (N05B, N05C, and N06A).

Dependent Variable	R	R Square	Adjusted R Square	Durbin-Watson
N05B	.954	.910	.905	1.043
N05C	.566	.320	.280	.558
N06A	.994	.989	.988	.424

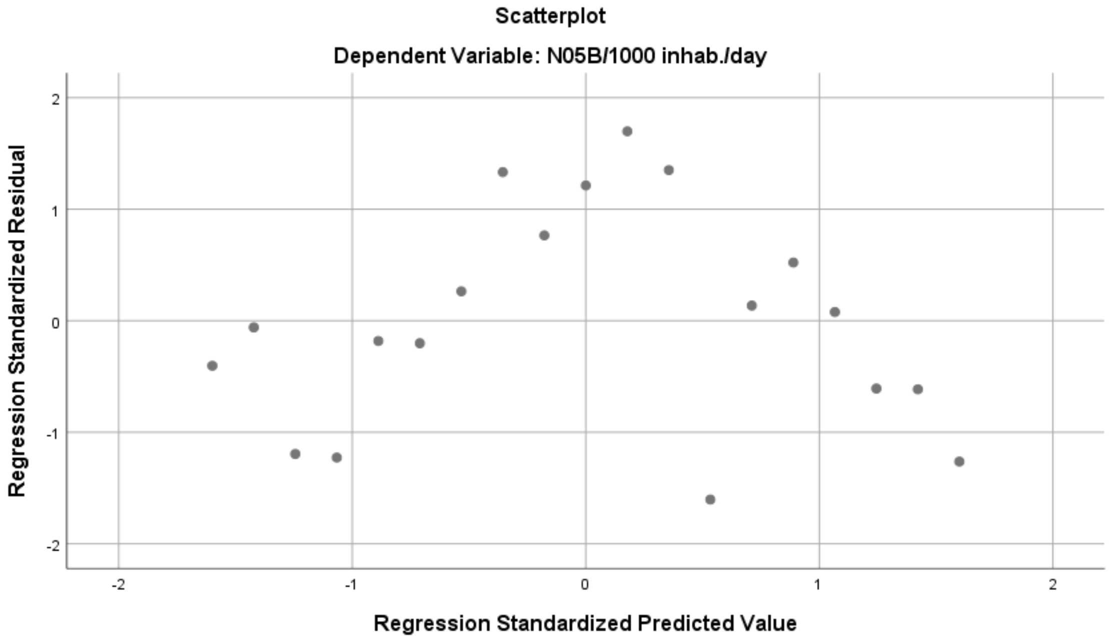

Figure S1. Linearity test output (zresid*zpred) - N05B.

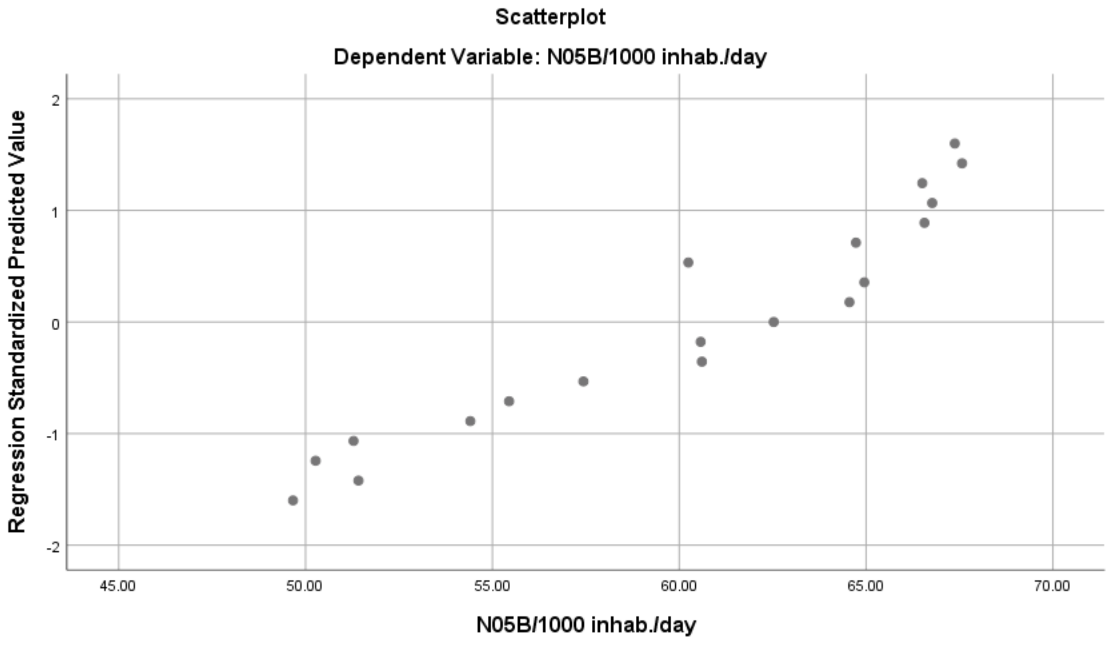

Figure S2. Linearity test output (zpred*dependnt) - N05B.

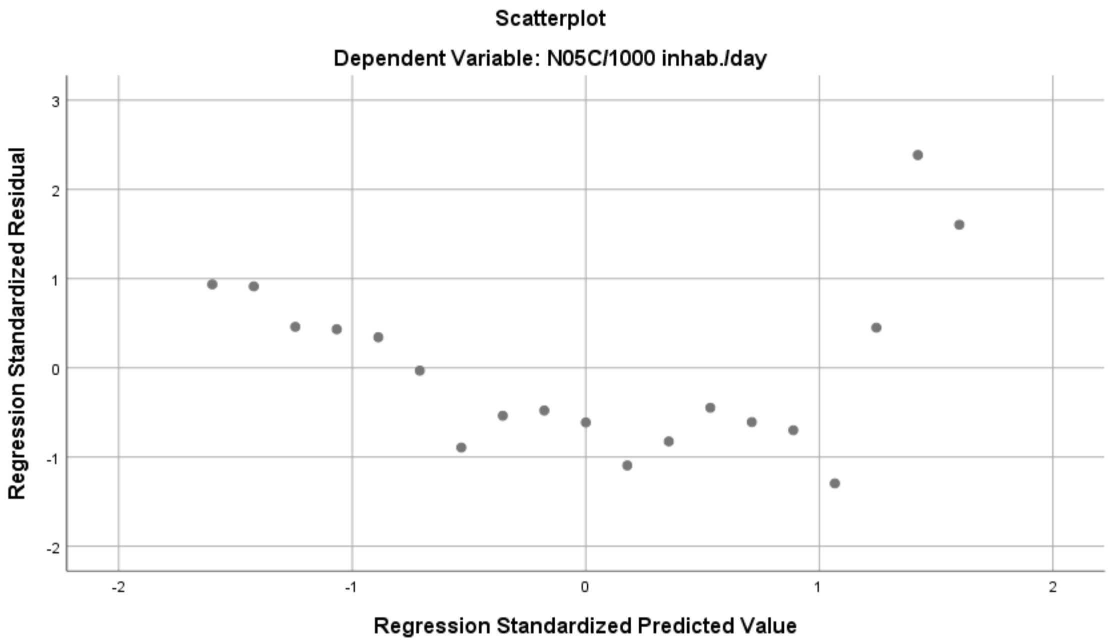

Figure S3. Linearity test output (zresid*zpred) - N05C.

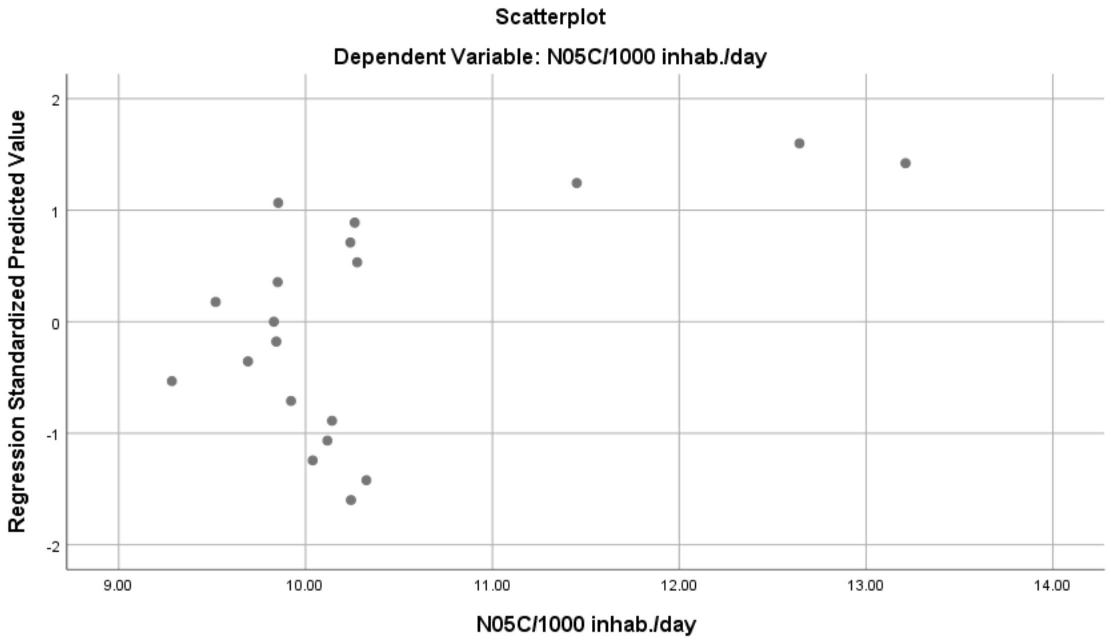

Figure S4. Linearity test output (zpred*dependnt) - N05C.

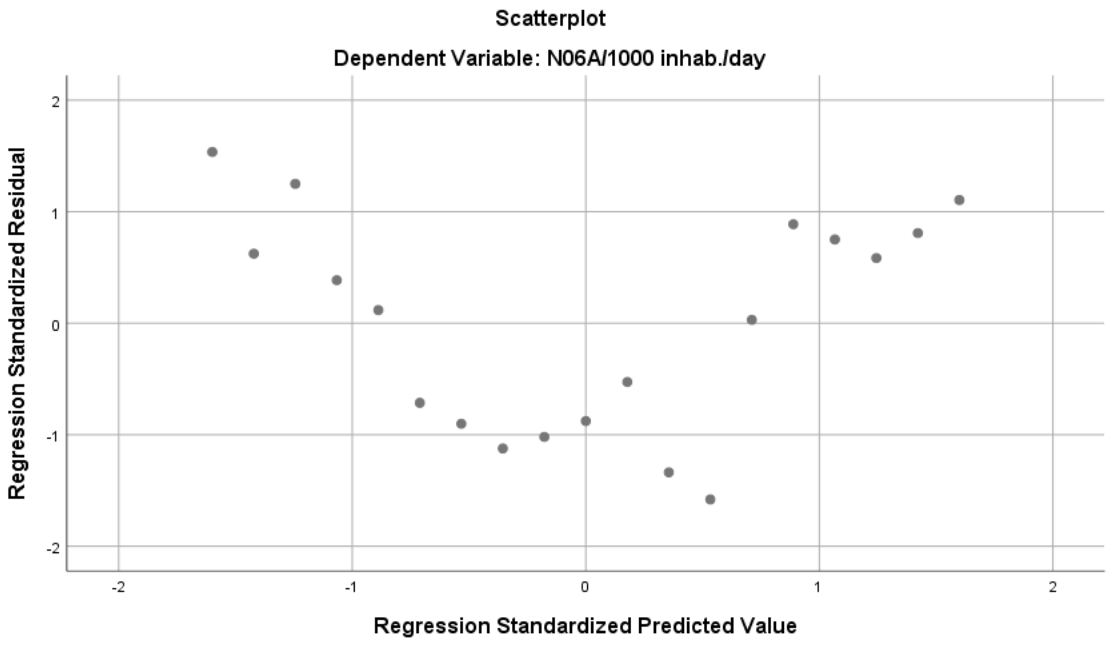

Figure S5. Linearity test output (zresid*zpred) - N06A.

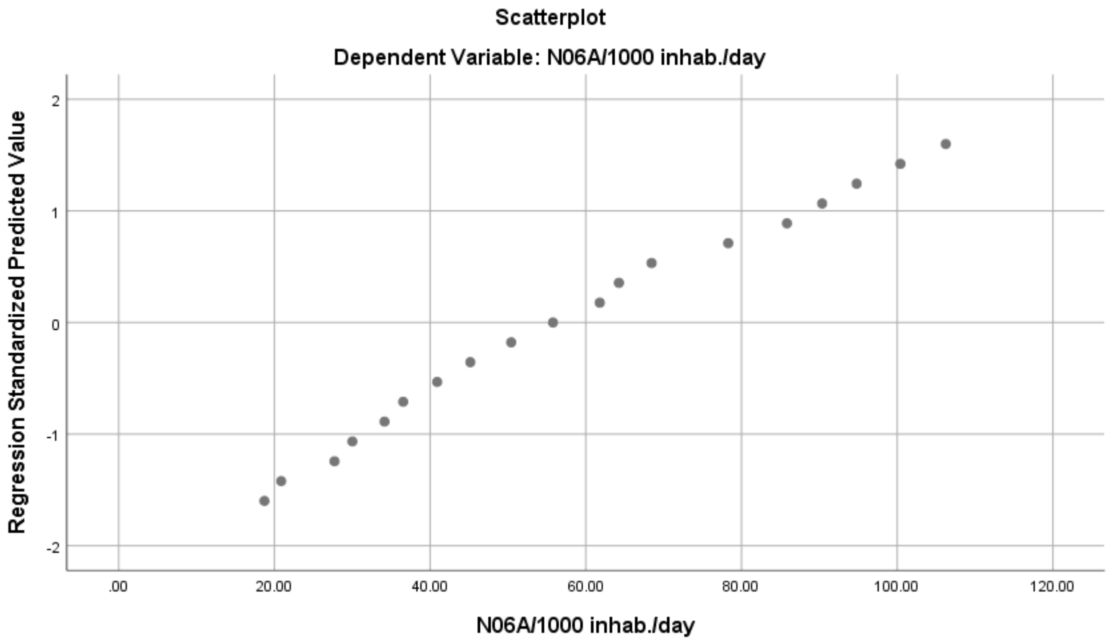

Figure S6. Linearity test output (zpred*dependnt) - N06A.

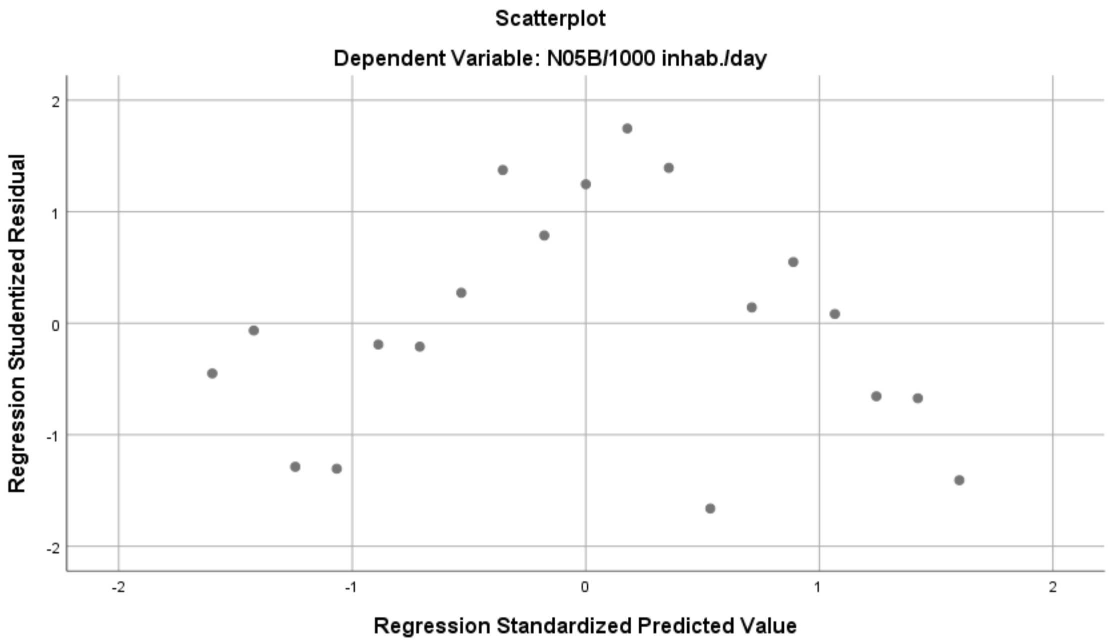

Figure S7. Homocedasticity test output (sresid*zpred) - N05B.

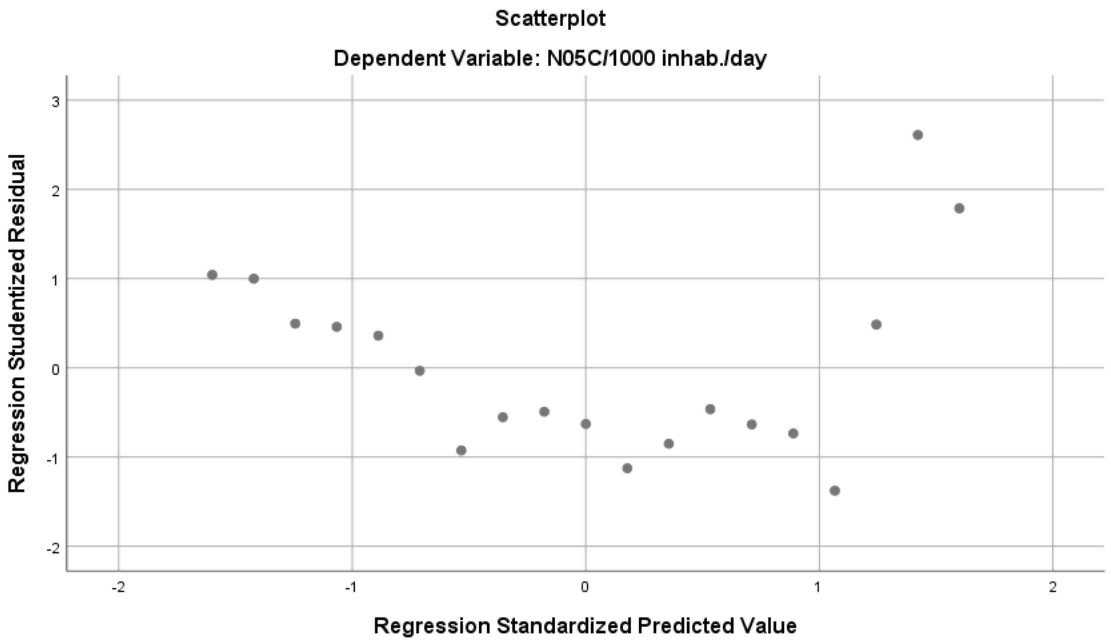

Figure S8. Homocedasticity test output (sresid*zpred) - N05C.

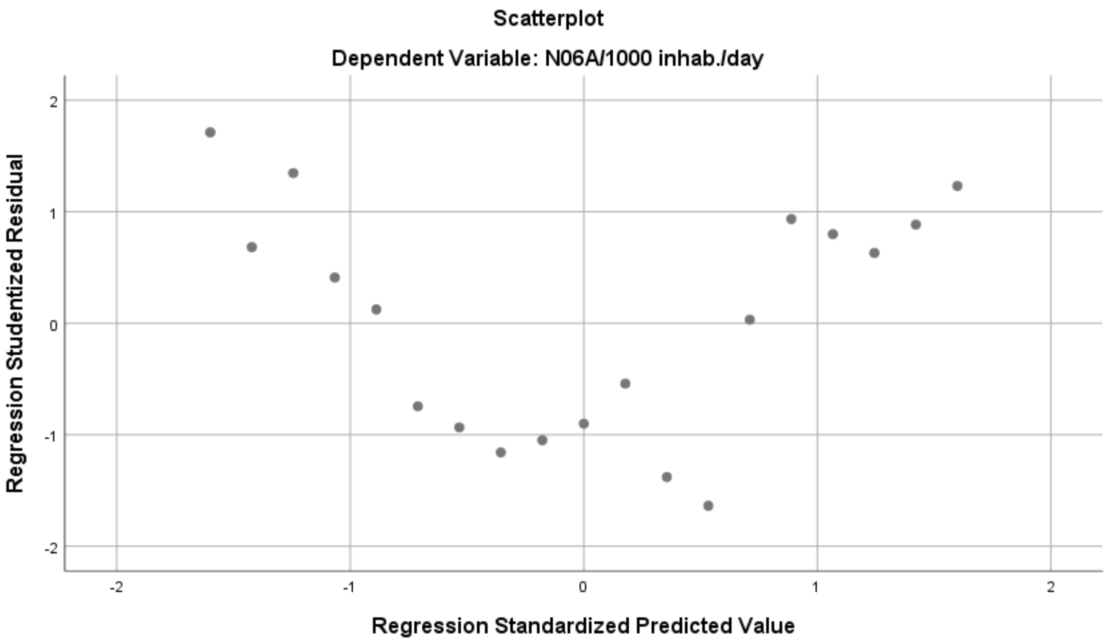

Figure S9. Homocedasticity test output (sresid*zpred) - N06A

Table S2. Normality tests output (N05B, N05C, and N06A).

Tests of Normality						
	**Kolmogorov-Smirnov[a]**			Shapiro-Wilk		
	**Statistic**	**df**	**Sig.**	Statistic	df	Sig.
Standardized Residual – N05B	.105	19	.200*	.962	19	.615
Standardized Residual – N05C	.204	19	.037	.922	19	.121
Standardized Residual – N06A	.147	19	.200*	.936	19	.223

*. This is a lower bound of the true significance.
a. Lilliefors Significance Correction
